# Partner’s emotional reaction to pregnancy mediates the relationship between pregnancy planning and prenatal mental health

**DOI:** 10.1186/s12884-021-03644-5

**Published:** 2021-02-27

**Authors:** Ashraf Kazemi, Maryam Ghaedrahmati, Gholamreza Kheirabadi

**Affiliations:** 1 Reproductive Health Department, School of Nursing and Midwifery, Isfahan University of Medical Sciences, Hezarjerib Av., Isfahan, Iran; 2 Reproductive Health Department, Isfahan University of Medical Sciences, Isfahan, Iran; 3 Behavioral Sciences Research center, Department of psychiatry, School of Medicine, Isfahan University of Medical Sciences, Isfahan, Iran

**Keywords:** Pregnancy, Emotional reaction, Depression, Anxiety, Mental health, Partner

## Abstract

**Background:**

An unplanned pregnancy may be followed by increased depression and anxiety. The aim of the present study was to evaluate the mediating role of partner’s emotional reaction to pregnancy (PERP) on the relationship between pregnancy planning and prenatal mental health.

**Methods:**

This cross-sectional study was conducted on 303 healthy Iranian pregnant women during their third trimester. The levels of depression and anxiety were measured using the Edinburgh Postnatal Depression Scale and the State-Trait Anxiety Inventory. The PERP score was also measured using a researcher-made questionnaire. The data were analyzed using the plug-in application PROCESS macro.

**Results:**

The results showed that PERP score was reversely related to pregnancy planning and prenatal depression and anxiety. The direct effect of the pregnancy planning on depression (c = −.05) and anxiety levels (c = −.02) were not significant; but the indirect effect of pregnancy planning on depression (Point Estimate = −.379, CI: −.523 to −.250) and anxiety levels (Point Estimate = −.560, CI: −.741 to −.385) with the mediating role of PERP were significant.

**Conclusions:**

The results indicated that the effect of pregnancy planning on prenatal mental health is mediated by PERP, and in unplanned pregnancy women need to receive positive reaction of their partners toward pregnancy so that they can preserve their mental health.

## Plain English summery

An unplanned pregnancy may affect women’s mental health. The mediating role of partner’s emotional reaction to pregnancy on the relationship between pregnancy planning and prenatal mental health was assessed in this study. This study was conducted on 303 healthy Iranian pregnant women during their third trimester. Using a validated tool the levels of depression, anxiety and partner’s emotional reaction to pregnancy were measured. The results showed that partner’s emotional reaction to pregnancy was reversely related to pregnancy planning and prenatal depression and anxiety. The results also showed that the effect of the pregnancy planning on depression and anxiety levels were not direct. However, the effect of pregnancy planning on depression and anxiety levels (indirect effect) were significant by mediating the partner’s emotional reaction to pregnancy. The results indicated that in unplanned pregnancy, women need to receive the positive reaction of their partners towards pregnancy so that they can preserve their mental health.

## Background

Mental disorders are among the most prevalent and important complications during pregnancy [1, 2], which can adversely affect the pregnancy outcome [3]. Adverse health behavior [4] and increased suicide attempts among pregnant mothers [5] are some of the related complications that can threaten the health of the mother and fetus. Furthermore, the negative effect of psychological disorders on marital relationships [6] may threaten the mental health of pregnant women. Therefore, many studies aimed to determine factors which affect the mental health of pregnant women. Studies have shown that women’s mental health during the pregnancy is associated with social support [7–9], marital relationships [10] and socioeconomic factors.

Moreover, unplanned pregnancy is known as a risk factor which can increase family conflict and depressive symptoms and decrease marriage harmony among partners [11, 12]. Some studies reported of increased risk of depression and anxiety following unplanned pregnancies [13, 14]. However, it is believed that even unplanned pregnancy can be associated with pregnancy acceptance [15]. Therefore, the relationship between pregnancy planning and women’s mental health during the pregnancy may be formed through the process of the couple’s relationship after an either planned or unplanned pregnancy.

The relationship between pregnancy planning and the factors associated with women’s mental health may explain this discrepancy. In a systematic review marital relationships and social support were reported to be important determinants for maternal mental health during the pregnancy [1]. Other studies have also shown that social support, especially the partner’s, is of great importance in maintaining the mental health of pregnant women [7, 16, 17]. Additionally, affecting the marital relations, the partner’s emotional support has important effects on the mental health of women during the pregnancy [8, 9] in such a way that some studies have reported that the partner’s emotional support is positively related to the increased marital satisfaction in women [18]. However, during the pregnancy, among the partner’s supportive behavior, his emotional reaction to pregnancy, that is, learning about his wife’s pregnancy or about pregnancy-related events such as the movement of the fetus or the growth of the abdomen as a result of the growth of the fetus, might be of special importance indicating the significance of the pregnancy to the partner.

In many women, occurrence of pregnancy is an event associated with great changes in sensations, emotions, and excitements [19] which are influenced by their familial relations [20] and can impact their mental health [9]. Moreover, among the social roles of women, the reproductive role is of special importance, as pregnancy for women is the realization of their gender roles so that decreased fertility in middle-aged women has been described as a sense of fruitlessness and unproductivity [21]. Previous studies indicated that many women consider pregnancy as the proof of their womanhood [22] and, thus, the partner’s lack of emotions about pregnancy may be perceived as indifference.

In a study on Japanese pregnant women’s satisfaction with family relations and their mental health, poor family relations was reported to have a direct association with increased morning sickness [20]. These findings show that physical signs, manifesting pregnancy, are of great importance for women. Therefore, in such societies, because of the emotional attachments between the partners, the partner’s confirmation of her fertility [23] might add to the importance of the partner’s emotional reaction to pregnancy and its effect on her mental health. According to these relationships, this study aimed to evaluate the partner’s emotional reaction to pregnancy (PERP) as a mediator of the relationship between pregnancy planning and the pregnancy-related psychological health.

## Methods

This cross-sectional study was conducted in Isfahan, Iran, from August 2017 to April 2018, approved by the Ethics Committee of Isfahan University of Medical Sciences. The study population included pregnant women in their third trimester receiving pregnancy care at health centers in Isfahan. The inclusion criteria were having a single pregnancy and not having any under treatment diagnosed medical, gynecological and mental disorders. The centers were selected from two networks in Isfahan using stratified random cluster sampling. Urban health networks organize health care centers services based on the Iranian health care system. In Isfahan, health services are organized by two health care networks. Therefore, two networks were considered as clusters and six health care centers covered by each network were selected randomly. Pregnant women who were referred to the centers were selected using convenience sampling. The inclusion criteria were evaluated by reviewing the women’s medical files. The sample size was determined by using single population proportion formula based on the assumptions of about 16% of prenatal anxiety prevalence from a previous study of the Iranian pregnant women [24] with a precision (margin of error) of 1.5% between the sample and population parameter. Considering a 95% confidence interval, the number of the participants was estimated to be about 303 pregnant women. After receiving the informed consent of the participants, their demographic and obstetric history (including parity, history of infertility, gestational age and pregnancy planning) and the pre-pregnancy history of depression prior were recorded.

### Measurements

The level of self-report depression was evaluated using the 10-items Edinburgh Postnatal Depression Scale designed with a 4-point Likert scale (0–3). The primary studies reported a cut-off point of 12 or higher as the depression index during pregnancy [25] and the validity for use with the Iranian population was approved with a Cronbach α of 0.79 [26]. The self-report anxiety level was measured using Spielberger Trait Anxiety Inventory, which measures trait anxiety. This questionnaire contains 20 items based on a 4-point Likert scale (1–4). The scores range from 20 to80, with the higher score indicating a greater trait anxiety. A cut-off point of scores ≥40 was coded as trait anxiety disorder (anxiety disorder). Furthermore, a depression level above 12 was considered as depression disorder [27].

The PERP was measured using a researcher-made 19-item questionnaire completed by the pregnant women. The items of the questionnaire were designed by performing content analysis on interviews conducted with 10 pregnant women or women with a history of pregnancy with the assistance of three psychologists. Based on the results, psychometrics of the initial version of a 19-item questionnaire with (with 3 inverse questions) was performed based on a 5-point Likert scale (1–5): strongly disagree (1), disagree (2), somehow disagree or agree (3), agree (4), and strongly agree (5).

For example, one of the items of the questionnaire was “observing fetal movement in my abdomen is interesting to my partner” or “the news of this pregnancy made my partner happy” or “I need something to happen to make my partner pay more attention to me”. The content validity ratio (CVR) and content validity index (CVI) of the questionnaire were calculated using the opinions of 10 experts. For the quantitative content validity, all 19 items remained because of a greater than 0.62 CVR and a more than 0.8 CVI.

For reliability assessment, a pilot study was performed on 14 eligible pregnant women and the tool was completed in two stages with a time interval of three weeks. The calculated Cronbach Alpha and intra-class correlation index were 0.92 and 0.99 respectively. The construct validity was assessed using exploratory factor analysis. Equity, a scree plot and parallel implementation of Monte Carlo test were used to finalize the items. A value of 0.4 was considered the minimum load factor. The Varimax rotation was applied to two factors indicating 52.01% of the observed variance. The equity values of 2 factors were larger than 1.6 for 25.341 and 6.860. Exploratory factor analysis (Table 1) led to a 19-item questionnaire and two subscales including affect to pregnancy events (APE) with 10 items and reaction to needs (RN) with 9 items. The score of PERP was the sum of the item scores of the questionnaire. The higher score, indicating the partner’s reaction to pregnancy, was more pleasant for women.

**Table 1:** The loading of the items of the partner’s emotional reaction to pregnancy questionnaire

Items	Load factors	
	Factor 1	Factor 2
***Affect to pregnancy events (10 Items)***		
Observing fetal movement in my abdomen is interesting to my partner	.667	
The news of this pregnancy made my partner happy	.637	
My partner’s emotional respond at the time of pregnancy diagnosis was satisfactory for me	.632	
Our health (me and baby) is important to my partner	.616	
My partner is constantly follow the growth of the fetus	.601	
I feel my partner makes an emotional connection with the fetus by touching my abdomen	.599	
Talking about the baby inside my belly is pleasant for my partner	.598	
My partner speaks about the fetus in a way as if he sees it	.511	
I feel that my partner appreciate the efforts I make for bearing pregnancy	.527	
My partner is worried about my and baby’s health	.517	
***Reaction to needs (9 Items)***		
My partner does not understand my physical condition and expects too much from me (Reverse)		.515
I feel that my partner does not care about me (Reverse)		.501
My partner has left me alone with all responsibilities of pregnancy (Reverse)		.672
It is important for my partner that I would not wear myself off by the housework		.611
Whenever I feel sad for pregnancy my partner do all in his power to help me		.553
My partner tries not to bother me during pregnancy		.677
I am sure that at the time of delivery my partner would accompany me to the maternity clinic		.665
My partner is by my side whenever I need him		.640
My partner is planning for the addition of the child to the family		.677

Statistical analysis was performed using SPSS software version 19. To test of the mediation effect, the plug-in application PROCESS macro v 3.4 was used. We created over 10,000 bootstrap samples to further test the indirect effects of PERP (95% confidence intervals).

The pregnancy planning (planed pregnancy: 1, unplanned pregnancy: 0) was the independent variable, the levels of depression and anxiety were the dependent variables and the level of the PERP score as an independent variable. To determine the potential confounding variables, Pearson and Spearman correlation coefficients between the depression and anxiety levels, women’s age, education, monthly income were calculated and the variables correlated with the main variables (*p* < 0.05) were considered as potential confounding variables. The variables correlated with depression, anxiety and PERP (and its subscales), entered the regression model as covariant.

## Results

The participants in the present study were 303 pregnant women with a gestational age of 28 to 36 weeks and a mean age of 32.56 years. Most of the participants in the study were multiparas (60.7%), with a secondary education and a monthly income level of under $ 1000 (Table 2). The results showed that the monthly income had a weak association with the depression (r = − 0.198, *p* = 0.002) and anxiety levels (r = − 0.199, p = 0.002) during pregnancy. Additionally, the link between the women’s age level (r = − 0.180, *p* = 0.003) and education (r = − 0.179, p = 0.003) and level of anxiety was significant and weak. The relationship between monthly income and partner’s reaction was also significant (r = .223, *p* = .001). Therefore, the monthly income, age and educational level as covariant variables entered the regression model.

**Table 2:** Descriptive profile of the participants (*n* = 303)

	Mean (SD) or Number (%)
Age (mean)	32.56 (5.33)
Educational level (%)	
Primary	13 (4.3)
Secondary	232 (76.6)
Higher	58 (19.1)
Monthly income ($)	
< 500	13 (4.3)
500–1000	216 (71.3)
> 1000	74 (24.4)
Gestational age (mean)	32. 6 (3.6)
Planed Pregnancy (%)	187 (61.7)
Depression level	9.1 (4.7)
Anxiety level	46.5 (7.8)
Partner’s emotional reaction (mean)	23.8 (16.5)
Affect to pregnancy events (mean)	13.3 (7.7)
Reaction to needs (mean)	10.5 (7.1)

Note: There was no missing data

The results showed that the PERP was related to pregnancy planning (*p* < .0001). Additionally, the results showed that, apart from these variables, women’s levels of depression (*p* < 0.0001) and anxiety (*p* = 0.002) had a significant inverse correlation with the PERP level (Table 3).

**Table 3:** The relations between antenatal mental health levels and partner’s reaction to pregnancy

	Dependent variables											
	Depression level				Anxiety level				Partner’s reaction to pregnancy			
	Adj r^2^ = .29; F = 21.08; p = <.0001				Adj r^2^ = .04 F = 2.87; *p* = .01				Adj r^2^ = .14; F = 11.05; p < .0001			
	Beta	Sig	CI 95%		Beta	Sig	CI 95%		Beta	Sig	CI 95%	
Age of women	.03	.55	−.06	.12	−.07	.22	−.58	.14	−.13	.02	−.07	−.77
Monthly income ($)	−.11	.03	−1.78	−.07	−.05	.38	−4.63	1.79	.17	.002	8.95	1.90
Educational level	.08	.11	−.09	.81	−.07	.27	−2.61	.74	.01	.91	−1.62	1.61
History of infertility	−.02	.64	−2.73	1.70	−.03	.59	−10.66	6.02	−.01	.81	−7.41	9.47
Pregnancy planning	.01	.67	−1.00	1.01	−.01	.92	−3.67	3.94	.32	<.0001	14.44	7.21
PERP (total)	−.52	< 0001	−.12	−.17	−.19	.002	−.07	−.29	–	–	–	–

*Abbreviation*: *Adj r*^*2*^ Adjusted r square, *CI* Confidence Interval, *PERP* Partner’s emotional reaction to pregnancy, *Beta* Standardized coefficients

**Fig. 1:**
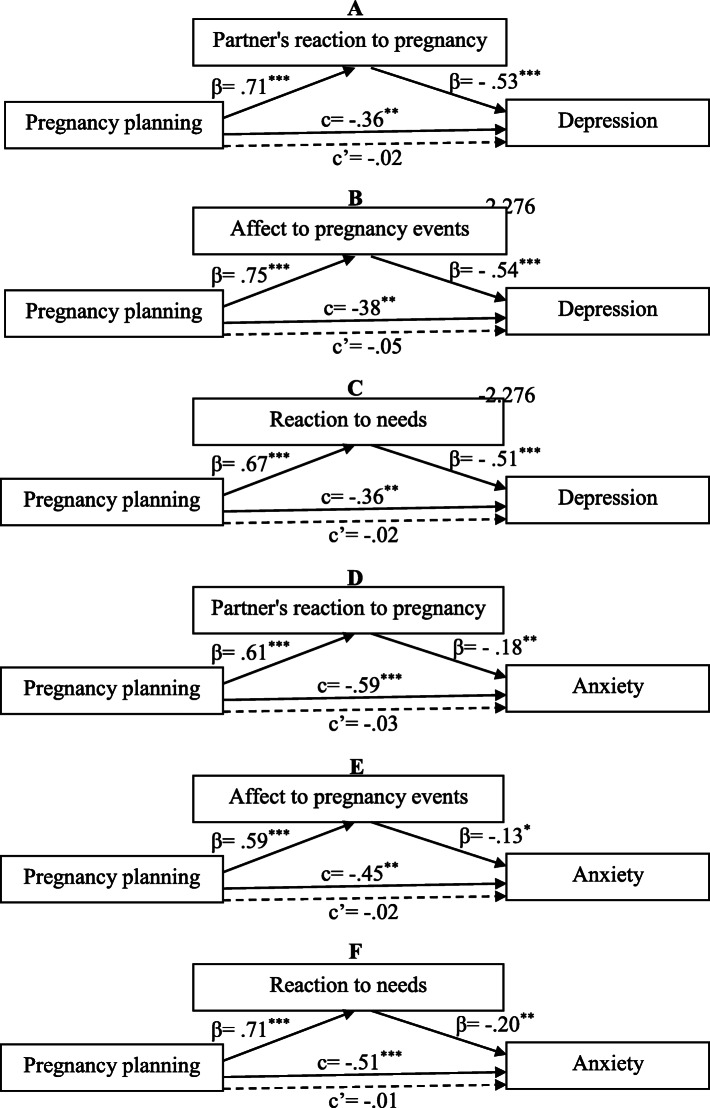
Mediation by partner’s reaction and its subscales to pregnancy of the association between pregnancy planning and depression (**a**, **b**, **c**) and anxiety (**d**, **e**, **f**). Abbreviations: c: standardized total effect; c’: standardized direct effect. *p < .05, **p < .01, ***p < .001

The levels of depression (β = −.21, *p* = .002, CI: −.14 to −.62) and anxiety (β = −.28, *p* = .003, CI: −.12 to −.55) were related to APE and RN respectively (−.53, *p* < .0001, CI: −.28 to −.38). The adjusted results for the level of education and monthly income showed that the indirect effect of pregnancy planning on depression and anxiety had been significant and inverse (Table 4). The direct effect of pregnancy planning on depression and anxiety was not significant, suggesting that the correlation of the pregnancy planning with depression and anxiety are completely mediated by the PERP and its subscales (Fig. 1).

**Table 4:**
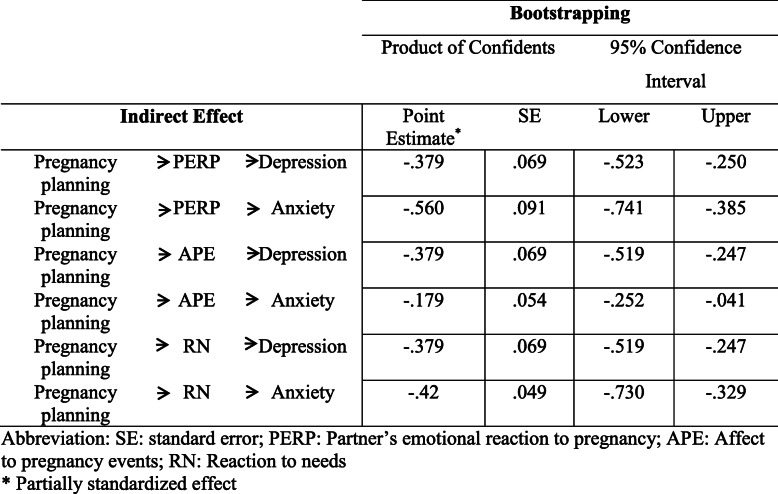
Indirect effects and specific indirect effects of pregnancy planning on depression and anxiety adjusted for age and monthly income.

## Discussion

The aim of the present study was to evaluate the PERP and its subscales as mediator of the relationship between pregnancy planning and the prenatal psychological health. Numerous studies have been conducted in evaluating the relationship between pregnancy planning and prenatal mental health, as well as the relationship between social support and prenatal mental health [28]. Nonetheless, as far as we know, the mediation of the PERP and the mental health of the woman during pregnancy have been evaluated for the first time.

The results showed that the desirable PERP, APE and RN decrease levels of depression and anxiety in the mother during pregnancy and, also, mediate the relationship between pregnancy planning and the prenatal depression and anxiety. These results indicate that to moderate the stressful pressures following unplanned pregnancy, the positive emotional reaction of the partner to the pregnancy is important.

Many studies have shown that the partner’s social support would decrease the woman’s levels of depression and anxiety during pregnancy [1, 23, 29] as well as the depression disorder [30];

Moreover, the PERP level is well-established as a major predictive factor for depressive and anxiety in pregnant women.

These findings complement the results of research showing an association between unwanted pregnancies and women’s psychological health during pregnancy [13, 14]. It also confirms the results of studies that have shown that marital relationship affects the prenatal psychological health [31, 32].

Another finding of this study showed a positive relationship between planned pregnancy and the PERP. The results also showed that the depression and anxiety levels in pregnant women were inversely related to the PERP level. These findings suggest that unplanned pregnancy reduce a PERP level and thus affects the prenatal mental health. However, the lack of a direct effect of the pregnancy planning on depression and anxiety indicates that if PERP is not decreased during unplanned pregnancies, an unplanned pregnancy will not increase the depression and anxiety of pregnant women.

Previous research has shown that unplanned pregnancies are accompanied by family disruption and a decline in the marital quality and the couples’ relationships; they may also be associated with a decrease in the partner’s emotional support and increase the risk of the women’s depression and anxiety.

It was reported that the partner’s emotional reaction to pregnancy as father was one of major themes in the fatherhood development process [33]. Thus, in the pregnancy of the woman, the father’s emotional reaction to pregnancy may be considered as a message of the fatherhood development and the partner’s approval of pregnancy; it may also affect the mother’s mental health. There have been reports showing the association of the unplanned pregnancy with an increased probability of not approving the pregnancy [34]. Also, some studies showed that the partner’s approval of pregnancy was associated with the improvement of the woman’s mental health and that the partner’s disapproval would lead to depression and anxiety during the pregnancy [2, 34].

Furthermore, the present study showed that although the PERP level was less frequent among women with lower economic status, where the PERP in these families was perceived as a desirable reaction, the levels of depression and anxiety decreased in among these women. Therefore, in families with lower socioeconomic status it is necessary to teach the partner the skill of expressing positive reactions in order to improve the mental health of pregnant women in unplanned pregnancy.

Although the partner’s approval of pregnancy was not directly measured in this study, the higher partner’s emotional reaction in the planned pregnancies, confirms this explanation. But the lack of direct effect of the pregnancy planning and women’s mental health may suggest that having a planned pregnancy is likely to affect women’s mental health, when is followed by the partner’s proper reaction.

Although the present study showed that, apart from the socioeconomic status, the mental health of pregnant women depended on their perception of the PERP, there were some limitations in interpreting the results that needed to be considered. The first notable limitation was that such factors as the partner’s personality characteristics could affect the form of the couple’s relationship and probably the PERP level and women’s mental health. Additionally, because the data on the PERP and the woman’s mental health during pregnancy were collected in a cross-sectional study, we could not establish the temporal relation between the two conditions. The women’s mental health could provoke a positive behavior in their partners and might have led to the partner’s positive emotional reaction to pregnancy. Depressed women also reported more negative responses by their partners. Besides, mental disorders might affect the partner’s perceived behavior in pregnant women that cannot be examined in cross-sectional studies. However, the measurement of relationship variables such as quality, length of relationship and other family-specific variables such as time since last child has been born (if there are other children) could improve future similar studies.

## Conclusions

The study showed that the partner’s emotional reaction to pregnancy mediates the relationship between the pregnancy planning and prenatal mental health indicating that the pregnant women need to receive their partner’s positive reaction towards pregnancy in order to preserve their mental health.

## Data Availability

Data and material are available on request from the corresponding author.

## Abbreviations

PERP: Partner’s emotional reaction to pregnancy
APE: Affect to pregnancy events
RN: Reaction to needs
CI: Confidence interval
